# A new dynamic *in vitro* modular capillaries-venules modular system: Cerebrovascular physiology in a box

**DOI:** 10.1186/1471-2202-14-18

**Published:** 2013-02-06

**Authors:** Luca Cucullo, Mohammed Hossain, William Tierney, Damir Janigro

**Affiliations:** 1 Cerebrovascular Research, Lerner Research Institute, Cleveland Clinic, 44195, Cleveland, OH, USA; 2 Department of Cellular and Molecular Medicine, Cleveland Clinic, 44195, Cleveland, OH, USA; 3 Department of Molecular Medicine, Cleveland Clinic Lerner College of Medicine, 44195, Cleveland, OH, USA; 4 Cleveland Clinic Lerner College of Medicine, 44105, Cleveland, OH, USA; 5 Current address: Department of Pharmaceutical Sciences Texas Tech University Health Sciences Center, 79106, Amarillo, TX, USA

**Keywords:** Neurological diseases, Shear stress, Venule, Atherosclerosis, Inflammation, Transmural pressure, Cerebral blood flow, Drug delivery, Organ-on-a-chip

## Abstract

**Background:**

The study of the cerebrovascular physiology is crucial to understand the pathogenesis of neurological disease and the pharmacokinetic of drugs. Appropriate models *in vitro* often fail to represent *in vivo* physiology. To address these issues we propose the use of a novel artificial vascular system that closely mimics capillary and venous segments of human cerebrovasculature while also allowing for an extensive control of the experimental variables and their manipulation.

**Results:**

Using hollow fiber technology, we modified an existing dynamic artificial model of the blood–brain barrier (BBB) (DIV-capillary) to encompass the distal post-capillary (DIV-venules) segments of the brain circulatory system. This artificial brain vascular system is comprised of a BBB module serially connected to a venule segment. A pump generates a pulsatile flow with arterial pressure feeding the system. The perfusate of the capillary module achieves levels of shear stress, pressure, and flow rate comparable to what observed *in situ*. Endothelial cell exposure to flow and abluminal astrocytic stimuli allowed for the formation of a highly selective capillary BBB with a trans-endothelial electrical resistance (TEER; >700 ohm cm^2^) and sucrose permeability (< 1X10^-u^ cm/sec) comparable to *in vivo*. The venule module, which attempted to reproduce features of the hemodynamic microenvironment of venules, was perfused by media resulting in shear stress and intraluminal pressure levels lower than those found in capillaries. Because of altered cellular and hemodynamic factors, venule segments present a less stringent vascular bed (TEER <250 Ohm cm^2^; P_sucrose_ > 1X10^-4^ cm/sec) than that of the BBB. Abluminal human brain vascular smooth muscle cells were used to reproduce the venular abluminal cell composition.

**Conclusion:**

The unique characteristics afforded by the DIV-BBB in combination with a venule segment will realistically expand our ability to dissect and study the physiological and functional behavior of distinct segments of the human cerebrovascular network.

## Background

The field of cerebrovascular research has created new and exciting opportunities for investigative and clinical studies. The challenge of reproducing the physiological characteristics and response of multiple brain vascular segments *in vitro* represents a critical biotechnological springboard for future mechanistic or preclinical studies. A realistic model of the brain circulation may significantly help understanding the mechanisms involved in the cerebrovascular response to a number of physiological and pathological stimuli. This, in turn will provide new strategies to accelerate the development on novel central nervous system (CNS) drug therapies and reduce the burden of major neurological disorders. As the research community recognize, mimicking the physiology of multiple vascular segments *in vitro* is a challenging task. An ideal cerebrovascular model should be able to reproduce the hemodynamic and cellular characteristics of each vascular segment. For example the vascular bed of brain microcapillaries selectively excludes most blood-borne substances from entering the brain and vice versa [1]. The venule segment is more permissive and allows leukocyte extravasation [2].

The barrier property of the cerebral vasculature depends on inter-endothelial tight junctions between adjacent endothelial cells that limit paracellular diffusion. At the BBB level, the endothelial cells are also characterized by low pinocytotic activity, lack of fenestrations, and unique expression patterns of trans-membrane transport proteins to regulate traffic into and out of the brain parenchyma [1]. Therefore, transit across the BBB involves translocation through the capillary endothelium by asymmetrically expressed carrier-mediated transport systems. These are responsible for passage of certain water soluble but biologically important substances such as glucose, mono-carboxylic acids, amino acids, *etc*. [1]. Furthermore, in addition to a physical and a transport barrier the BBB endothelium acts as a metabolic barrier. This function is mediated by a BBB-specific cytochrome P450 enzymes that catalyze the biotransformation of lipids and steroidal hormones, as well as xenobiotics For example, the antiepileptic drug undergoes brain-specific metabolism in addition to its known conversion by liver P450 enzymes [3].

BBB endothelial cells are surrounded by astrocytic end feet processes sharing a basal lamina, and enveloping > 99% of the BBB endothelium [4-6]. Astrocyte interactions with the cerebral endothelium modulate BBB function, regulate protein expression, facilitate endothelial differentiation and play a major role in the expression and maintenance of functional inter-endothelial tight junctions as well as of other BBB properties [7].

### Flow plays a crucial role in modulating BBB functions

The exposure to physiological shear stress (SS) also plays a critical role in modulating BBB functions and facilitating the differentiation of vascular endothelial cells into a BBB phenotype [8-10]. Flow across the apical surface of the vascular endothelium activates a number of mechanosensors (e.g., integrins, *caveolae*, G proteins, and ion channels) [11-13] which transduce physical stimuli into biochemical signals. Despite the variety of potential mechanosensors present on the luminal side of the endothelial cell membrane one of the major common downstream effect is the activation of extracellular-signal-regulated kinases 1/2 (ER [14] K1/2). These are pleiotropic modulators of the cell physiology and play an important role in the control of the expression of gene involved in the regulation of cell division, apoptosis, cell differentiation and cell migration [9,10,13,15-18]. Interestingly, expression of these genes in endothelial cells is under the control of shear stress [10,13].

### Rheological and architectural characteristics of distal brain venules

The architecture and cellular milieu of these vessels are remarkably different from that of the BBB due to the existence of mural cells (where smooth muscle cells start gradually appearing in venules with a diameter > 30 μm up to ≅ 50 μm) [19] and perivascular spaces. This affects the organization of the inter-endothelial tight junctions [20], which leads to the formation of a significantly less selective vascular bed than BBB capillaries [21]. There is general agreement that, venular endothelial cells are exposed to a significant lower level of SS (between 1.5 to 4.5 dynes/cm^2^) than those forming the capillary vascular bed. Direct measurements of shear stress values in BBB vessels and their venular counterparts are lacking, but given our previous work showing a direct effect of shear on the physiological and functional properties of the vascular endothelium it is reasonable to assume that capillary vs. venules differ in the properties in party owing to shear levels.

## Results

### Modular dynamic capillary-venule *in vitro* system: Physiology in a box

**Figure 1:**
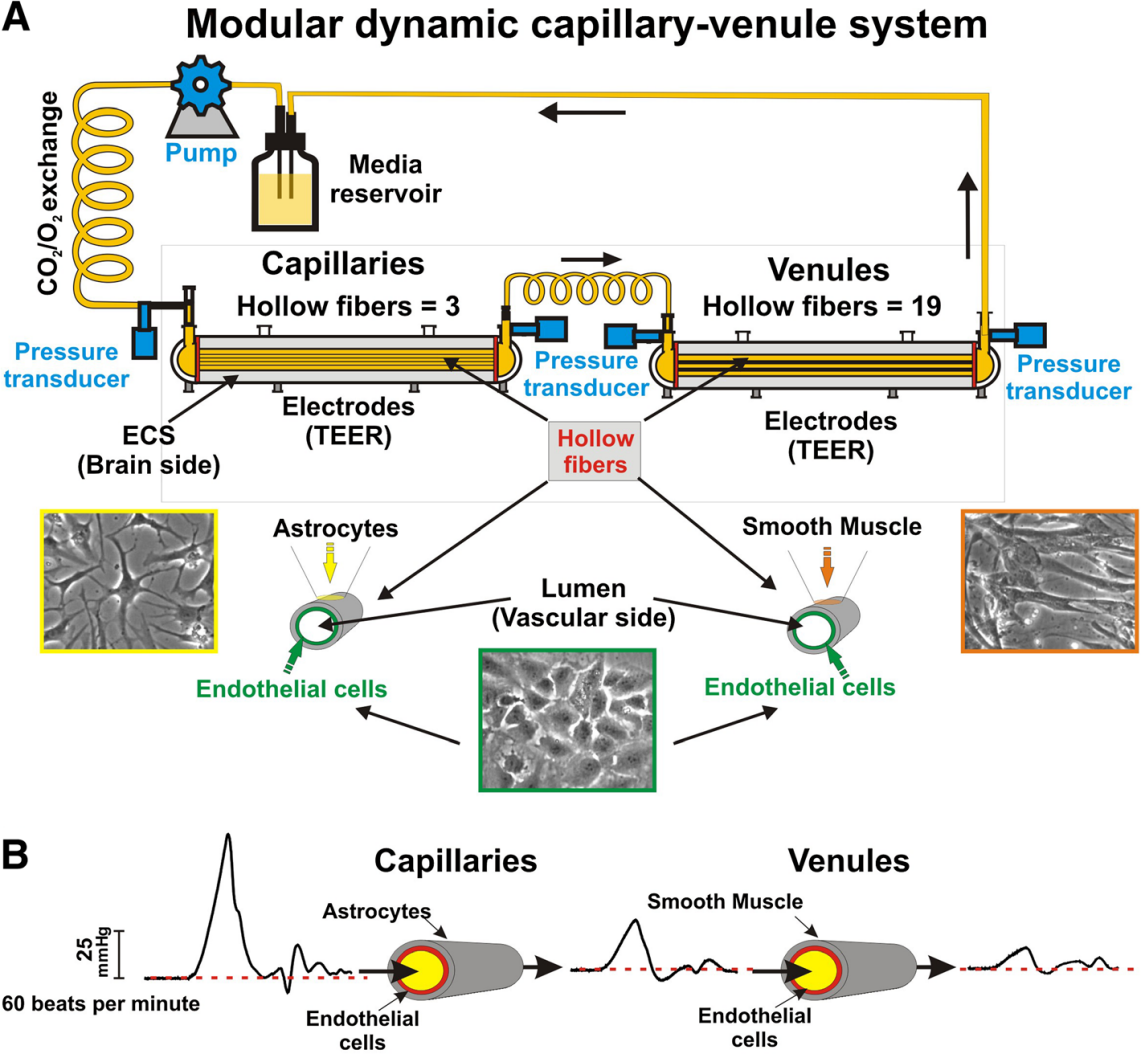
**Schematic outline of the DIV capillary**-**venules model.** Note how the system recapitulates both rheological and cellular characteristics of the corresponding *in vivo* cerebrovascular segments.

One of the major limitations of current vascular *in vitro* models is their inability to mimic the functional characteristics and response of multiple vascular segments within the cerebrovascular network. To address this problem we have developed a new dynamic *in vitro* model that recapitulates the hemodynamic, metabolic and functional characteristics of capillary and post-capillary vessels of the human brain vascular network. The modular assembly of the system (Figure 1A) originated from a serial combination of capillary and venule modules. In this configuration, a fully established BBB module influences its respective venule module through gas permeable silicon tubing connecting the respective luminal compartments. Each module reproduces as closely as currently possible the cellular composition of its corresponding vascular segment *in vivo*. In the capillary module, luminal human primary brain microvascular endothelial cells were co-cultured with abluminal human astrocytes to mimic the cellular milieu forming the BBB microcapillaries *in vivo*. In the venules module, the abluminal glial cells were replaced by human vascular smooth muscle (HUSMC) as observed in distal post-capillary segments of the cerebral vessels [22]. A peristaltic pump within the system generated a pre-capillary high flow velocity input characterized by an arterial systolic-like blood pressure of ≅ 70mmHg (see Figure 1B). Within the system medium flow moves through a gas permeable silicon tubing allowing the exchange of oxygen and CO_2_ with the external environment before entering into the first module (capillary system). The number of hollow fibers in the capillary (n=3) and venule (n=19) modules were determined to mimic the rheological characteristics (transmural pressure, flow rate and shear stress) of the corresponding cerebrovascular segments *in vivo*[23]. For the experiments shown herein, the flow rate was between 4.6 and 5.3 mL per minute. Note the significant pressure reductions observed when flow passed first through the capillary and then through venule segments (see Figure 1B). Our data showed that the transmural pressure and the shear stress were consistent with the corresponding *in vivo* observations (see Table 1). Furthermore, a computer controlled pumping mechanism allowed us to reproduce a broad range of perfusion scenarios, each characterized by different levels of shear stress, intraluminal pressure, pulsatile rate to reproduce heart beats/min.

**Table 1:** **Side**-**by**-**side**, **comparison between rheological parameters measured *in vitro *versus *in vivo***

**Blood Pressure**(**mmHg**)	***In vivo***	***In vitro***
Pre-capillaries	60	70.1 ± 0.2
Capillaries	25	25.5 ± 0.1
Venules	12-15	11.8 ± 0.4
**Shear stress(dyne/cm**^**2**^**)**		
Capillaries	5-23	16.3
Venules	3 ± 1.5	2.6

### The capillary-venule *in vitro* system can mimic the rheological characteristics of the corresponding vascular segments *in vivo*

**Figure 2:**
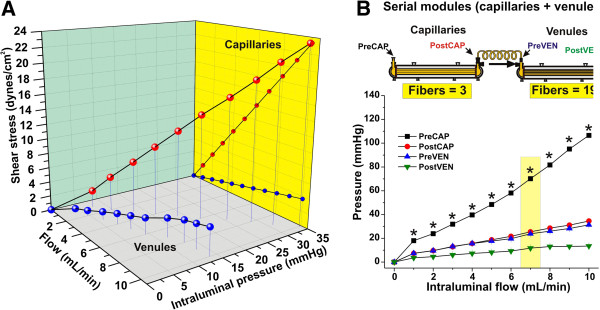
**Rheological characteristics of the DIV capillary**-**venule system.** Panel **A**: Hemodynamic profile of capillary and venule segments in respect to perfusion. Panel **B**: Note that the presence of inter module connector between the capillary and venule lumens did not alter the rheological profile of flow. The asterisk “*” indicates a statistically significant difference in transmural pressure between capillary and venules (n=4; p<0.05).

Figure 2A shows changes that occurred in the hemodynamic profile (transmural pressure and shear stress) of capillary and venule segments in respect to perfusion. Note that increases in the perfusion rate determined a significant proportional increase in both shear stress (dynes/cm^2^) and intramural pressure (mmHg) in the capillary segment (Figure 2A –left panel; red dots). Table 1 shows a comparison between *in vivo* and *in vitro* parameters. Changes in the corresponding shear stress and intramural pressure measured in the venule segments are significantly less evident (Figure 2A –left panel; blue dots). Note (see Figure 2B) that the tubing connecting the luminal output of the capillary module to the venules did not affect the rheological characteristics of flow. This is shown by comparing post-capillary segment (post CAP) to pre- venous (Pre VEN) pressure values. Therefore, from a hemodynamic standpoint, the two modules behaved as a contiguous vascular system, which however, exhibited distinct capillary and post-capillary rheological features characteristics of the corresponding segments *in vivo*.

### Vascular integrity and permeability characteristics of capillary and venules *in vitro*

**Figure 3:**
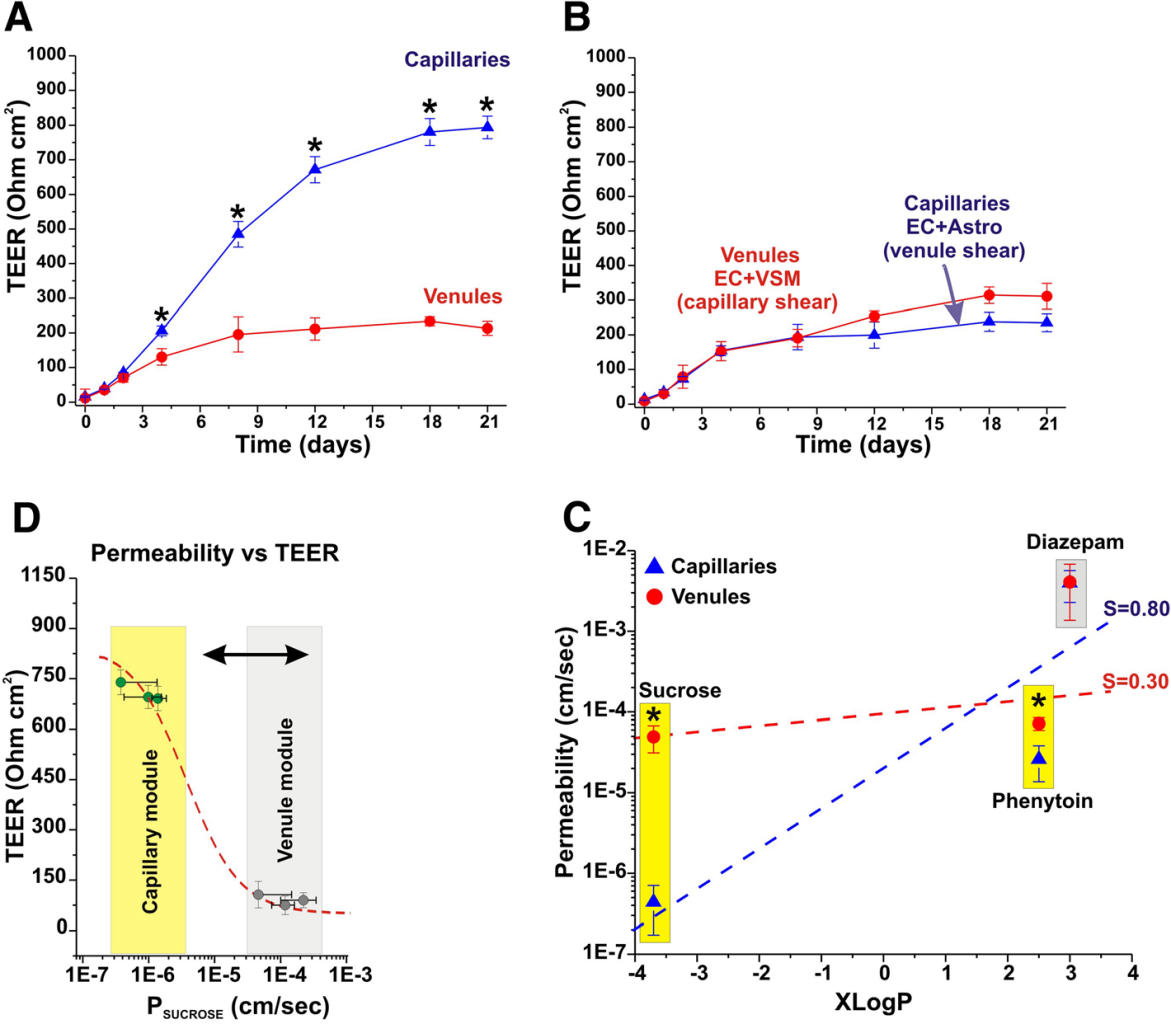
**Side by side comparison between *in vitro *capillary and venule vascular beds.** Panel **A**: Note how the capillary system allows for the formation of a very stringent vascular bed (high TEER) in comparison to venules (low TEER). (Panel **B**). Note also that capillary segments established under venules level of shear stress and venules module exposed to capillary shear stress levels formed a comparable low stringent barrier suggesting that both abluminal astrocytes and high shear stress levels are necessary to develop a tight vascular bed (Panel **C**) TEER and sucrose permeability correlation in capillaries and venules modules. The sigmoid curve symbolize the ideal correlation between TEER and permeability previously determined by us [55]. Note the difference of ≈ 2 order of magnitude between capillaries (less permeable) and venules (most permeable). The more stringent vascular bed formed in the capillary module can discriminate drug permeability based on the octanol-water partition coefficient (XlogP) with a significantly higher degree of selectivity than venules (Panel **D**). The asterisk “*” indicates a statistically significant difference (n=4; p<0.05).

[24] between capillaries and venules *in vitro* (see Figure 3C). Furthermore, Figure 3B shows that astrocytes, in presence of venous perfusion flow, are not sufficient to induce a high TEER and low paracellular permeability. However, astrocytes are necessary for the development of a tight barrier since when venule modules were exposed to capillary levels of shear stress we did not observe any significant increase in TEER.

A functional capillary/BBB model must be able to discriminate the permeability to molecules based on their oil/water partition coefficient. In addition, a capillary-venule model must be able to differentiate the permeability to the same compound between each segment. In fact, where the tightness of the vascular endothelium is a determining factor for the permeability the capillary vascular bed will impede the passage of polar molecule more effectively than that of the venules. To validate our model we performed permeability tests across each module using three different classes of compounds: 1) Sucrose (a well-established paracellular marker); 2) Phenytoin (an anti-epileptic drug and a moderate substrate for multidrug transport systems, e.g., P-glycoprotein); 3) Diazepam (a benzodiazepine that crosses the BBB by passive diffusion). Permeability of each compound was calculated for each segment (capillary and venules) by integrating the area under the ECS and lumen data points (AUC) between time 0 and time (t) = 20 minutes (min) according to the equation described in the methods section [25].

Figure 3D shows the results of the permeability measurements in the capillary and venule segments. Permeability to Diazepam (P_DIAZEPAM_; logP ≅ 3) measured in the capillary module was comparable to that observed in the venules (3.97 × 10^-3^ ± 1.70 cm/sec and 4.09 × 10^-3^ ± 2.72 cm/sec respectively). Phenytoin permeability (P_PHENYTOIN_; logP ≅ 2.5) measured in the capillary was 2.59 × 10^-5^ ± 1.22 cm/sec versus 7.20 × 10^-5^ ± 1.26 cm/sec in the venule. Sucrose (the least lipophilic compound tested; logP ≅ -3.7) permeability (P_SUCROSE_) was 4.39 × 10^-7^ ± 2.68cm/sec and 4.93 × 10^-5^ ± 1.80cm/sec in the capillary and venule segments respectively.

### Vascular response to a hyperosmolar agent

**Figure 4:**
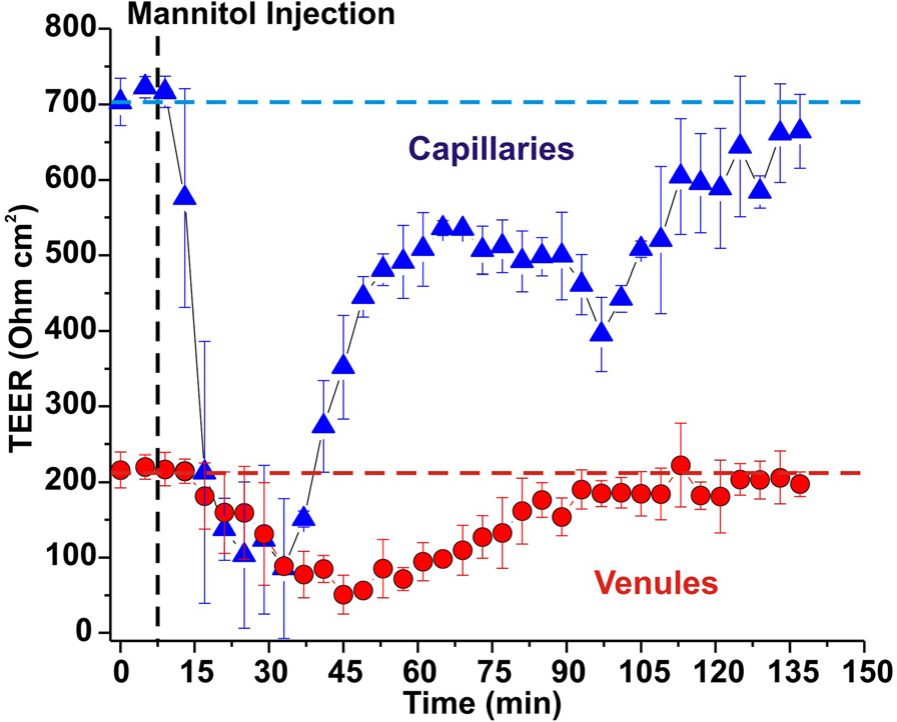
**Functional characterization of the DIV capillary**-**venule system.** Hyperosmolar opening of the BBB in DIV models was assessed by real time measurements of TEER. Similar to what observed *in vivo* the differential magnitude and the transient nature of the vascular opening is indicative of the formation of capillary and venule vascular beds that closely mimic the physiological response of the corresponding cerebrovascular segments in situ.

Intracarotid infusion of hyperosmolar [1.6 M] mannitol, a cell-impermeable and non-toxic polyalcohol, has been to previously used to reversibly disrupt the BBB *in vivo*[26] to facilitate the passage of chemotherapic drugs (e.g., methotrexate) in the treatment of malignant brain tumors [27]. The osmotic opening of the vascular endothelium is a non-energy-dependent mechanism [28] solely mediated by dehydration of endothelial cells, cerebrovascular dilatation, and contraction of their cytoskeleton protein structures. We tested the effect of hyperosmolar mannitol injection on vascular integrity in the capillary and venule modules. The intravascular compartment of DIV capillary-venule system was perfused for 120 sec with hyperosmolar media containing mannitol [1.6 M]. The flow rate used was 1 mL/min. TEER was measured to assess real-time changes of vascular integrity in the capillary and venule modules (Figure 4). We observed a transient (≈ 37 minute ± SEM 4) loss of vascular integrity (TEER <400 Ohm cm^2^) in the capillary module. This was paralleled by a loss of vascular integrity in the venule module which was significantly longer in duration (≈ 55 minute ± SEM 5) although less significant in magnitude.

### Bioenergetic metabolism of capillary and venule cerebrovascular segments

**Figure 5:**
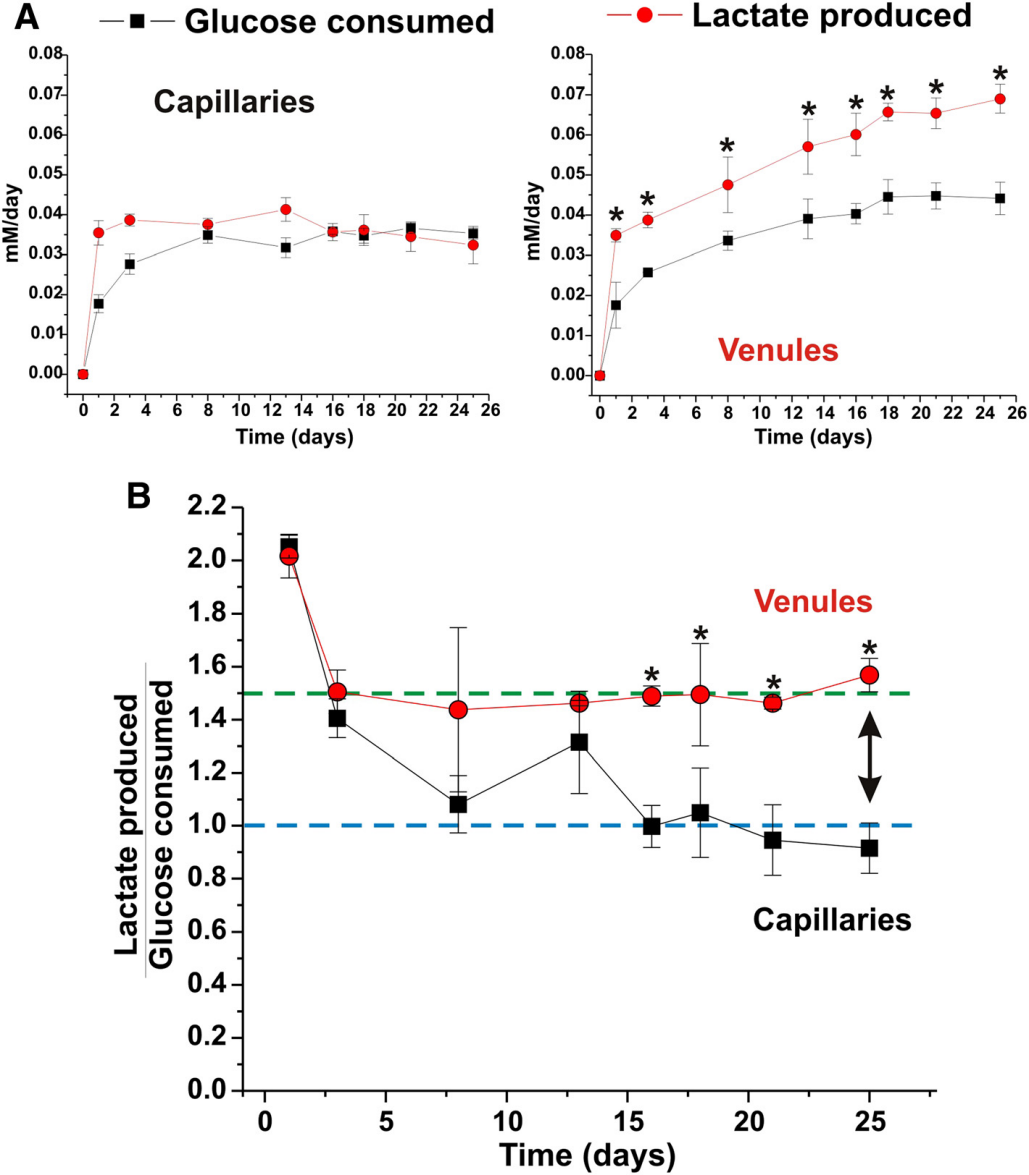
**Differential bioenergetic behavior of *in vitro *capillary and venule vascular systems.** Panel A: Glucose consumed versus lactate produced over time in capillary and venule modules. Note the significant difference between the glucose consumption and lactate production ratio (R) in the capillary module (R≅1) and in the venule (R≅1.5). This indicates that in contrast to venule segments, the brain capillary cellular elements have increased propensity towards the use of aerobic-based glucose metabolism. The asterisk “*” indicates a statistically significant difference (n=4; p<0.05) versus parallel systems established under static conditions.

The assessment of cell metabolism (glucose consumption/lactate production) provides important information on the bioenergetic mechanisms (aerobic-anaerobic) used by brain vascular segments. Figure 5 shows changes in glucose consumption and lactate production in the vitro capillary and venule segments. Note that during anaerobic respiration each molecule of glucose is converted into 2 lactate molecules (glucose consumption/lactate production ratio = 2) while during aerobic respiration glucose in totally converted into CO_2_ and H_2_O. In the capillary segment (Figure 5A), lactate production-glucose consumption ratio (1 ± SEM 0.08) was suggestive of a bioenergetic behavior favoring oxidation. In contrast, in the venule segment (Figure 5B) we measured a lactate production-glucose consumption ratio of 1.5 ± SEM 0.14, thus suggesting that in the presence of reduced vascular shear stress (in comparison to the capillary segment) the cellular bioenergetic demand is largely fulfilled through anaerobic glucose metabolism [10]. These preliminary results need larger scale validation and experiments where cell variables (e.g., vascular smooth muscle vs. astrocytes) are interchanged. This is evident in Figure 5C which shows the side-by-side comparison of the glucose consumption-lactate production ratios between capillary and venule segments established in the DIV system.

## Discussion

Hemodynamic alterations as well as pro-inflammatory and exogenous stimuli can adversely affect vascular integrity and lead to the pathogenesis and/or progression of a number of major neurological disorders [5,29-31]. *In vitro* studies are set to support and facilitate our understanding of the mechanisms involved in the physiological and pathological modulation of cerebrovascular functions. This is of critical importance for the development of novel strategies aimed at reducing the burden of CNS disease associated with brain vascular impairments. Other *in vitro* systems have attempted to reproduce the physiological and functional characteristics of capillary or venular segments of the cerebrovascular system (e.g., [32,33]). However, these models lack the ability to reproduce the environmental cues (e.g., hemodynamic stimuli) to which these vascular beds are exposed *in vivo*.

The use of hollow fiber technology allowed us and others to establish the first quasi-physiological *in vitro* BBB models [34-39] which was then further humanized [40] to reproduce not only healthy BBB properties but also properties of multiple drug resistance and leukocyte extravasation [2,24,41]. This technology allows to develop the first artificial interlinked brain capillaries and venules segments, which retain their distinct vascular properties. As shown in Figure 1 the pumping mechanism generates arterial high velocity flow with a systolic blood pressure range (80 to 300 mmHg) comparable to *in vivo*. A dramatic drop of perfusion velocity and transmural pressure occurs when flow enters the capillary segment generating a vascular shear stress comparable to what has been reported for non-BBB vessels *in vivo*[42]. After leaving the capillary module, the medium flow enters into the venule module were perivascular astrocytes were replaced by smooth muscle cells to more closely mimic the *in situ* cellular milieu of this vascular segment. At this level systolic transmural pressure is low (see Figure 2) and flow is characterized by low shear stress (≅ 3 dynes/cm^2^; see also Table 1). It is important to underscore again that direct measurements of shear levels in brain microvessels is lacking and that the values used in this manuscript are extrapolations from other vascular beds *in vivo*[42].

The DIV capillary-venule model not only mimics the rheological characteristics of the corresponding brain vascular segments but also their functional and physiological properties (e.g., Table 1). In agreement with previously published studies BBB capillaries *In vivo* are characterized by high TEER, lack of paracellular pathways, low permeability to polar molecules (e.g., paracellular markers) and a selective permeability that (in the absence of specific extrusion mechanisms) reflects the lipophilicity of the specific substance [1]. Our results have clearly shown that, similar to *in vivo*, the artificial capillary vascular bed was characterized by a high TEER (see Figure 3A and 3D); low permeability to the paracellular marker sucrose, and was capable of selective permeability reproducing the *in vivo* rank order of the tested substances (see Figure 3C) [43]. Overall, the relationship between lipophilicity and permeability found in the DIV-capillary module was similar to that reported by others *in vivo*[44].

Venules are characterized by a significantly more permissive vascular bed with a lower TEER and a reduced ability to provide a barrier to the passage of polar molecules than capillaries [45]. In this respect, the DIV-venules showed lower TEER, and a reduced selective permeability (see Figure 3A, 3C and 3D). Our findings also suggest that abluminal astrocytes and high shear stress are both necessary to establish a tight vascular bed [4,6,10,37,38]. In fact, when the capillary modules were established under venular level of shear stress and vice versa, (venule modules established under capillary-like shear stress levels; see Figure 3B) no TEER increase suggesting the formation of stringent vascular bed was observed.

BBB opening following exposure to hyperosmotic mannitol is common clinical procedure used to enhance chemotherapeutic drug penetration into the CNS to treat patients with metastatic or primary brain tumors [46]. Hyperosmotic opening of the BBB is mediated by vasodilatation and shrinkage of cerebrovascular endothelial cells (and perhaps glia), with widening of the inter-endothelial tight junctions to an estimated radius of 200 Ǻ. This provides paracellular pathways previously lacking that can facilitate the passage of substances across the brain capillary endothelium [21,27,28,47]. Our results (see Figure 4) demonstrated that loss of vascular integrity of in the DIV capillary-venules model mimics the expected physiological vascular response to hyperosmotic agents (magnitude and duration) of the corresponding vascular segments *in vivo*. The tightness of brain capillary vascular beds is the most dependent upon the tight junctions’ (TJ) ability to seal the space between adjacent endothelial cells. The presence of tight junctions between endothelial cells in our capillary model was demonstrated by electron microscopy [38] and confirmed by functional assays that measured for example permeability to K ions [37] The mechanisms of osmotic blood–brain barrier disruption is believed to depend on endothelial cell shrinkage. This may be caused by the exposure to a hyperosmolar environment, efflux of water from the endothelial cell and subsequent cytoskeletal rearrangement. The latter may cause stretching of TJ and widening the interendothelial space. The temporary formation of paracellular routes of entry across the BBB as demonstrated by the monophasic TEER decrease.

The effect of mannitol was strikingly different in the venule compared to capillary modules. Recovery was delayed and overall disruption achieved was less notable at the venular level; the latter may be due to its more prominent paracellular pathway. There is no clear-cut explanation for the delayed recovery of venular TEER after osmotic challenge. However, we would like to underscore that when we performed experiments *in vivo* (rat, pig; intracarotid injections of mannitol) we found that at 30’-1 hr. intervals white matter venules retained blood–brain barrier disruption properties (measured by different tracer means) while gray matter small vessels (capillaries) were, at this time point, “intact”. The fact that we can reproduce this finding *in vitro* suggests that the persistence of disruption in larger vessels, and venules as seen in white matter, is due to intrinsic properties rather than vascular access issues, or parenchymal influences. It is also critical to address the issue of molecular mechanisms of osmotic disruption and recovery of TEER. For example, are TJ protein involved in a transcriptional vs. positional way? Are other mechanisms such as transcellular access relevant? We believe that our findings require additional studies, and that only a side-by-side comparison of our *in vitro*/*in vivo* will elucidate this. Finally, it is striking that a clinically relevant procedure such as osmotic BBBD has never been mechanistically explored to show what truly are the mechanisms underlying increased permeability after intrarterial mannitol.

Oxygen delivery to the CNS is another important function bestowed upon the brain vessels. Recent studies have shown that PO_2_ values increase from the post-capillary venules to the distal vessels of the cerebrovascular network and by contrast, measurements of the radial gradients are consistent with an increase oxygen loss [48]. These data support the hypothesis that venules may indeed play a critical role in oxygen delivery. These findings are consistent with our data related to the metabolic behavior of capillary and venule segments. In fact, the highly oxygenated blood from the arterial circulation reaching the brain microcapillaries allows the BBB endothelium to make use of the highly efficient aerobic-driven metabolic respiration to meet the cellular bioenergetic demand and to use less glucose in the process thus maximizing that delivered into the brain (see Figure 5A). On the other hand, if oxygen delivery to the brain primarily occurs at the venular level as suggested by recent studies [48,49] than a non-oxidative (anaerobic) metabolic behavior (see Figure 5B and 5C) would minimize vascular oxygen consumption leaving more oxygen available for brain delivery. Furthermore, recent studies have clearly shown that shear stress plays a key role in the modulation of bioenergetic metabolism of vascular endothelial cells favoring the utilization of the more energy rewarding aerobic pathway. This perhaps also suggests an additional link between shear stress/flow and vascular functions of the different cerebrovascular territories. However, additional and more specific studies will be necessary to validate this hypothesis and to understand the underlying mechanisms.

A limitation of our study concerns the lack of pericytes to the abluminal mixture of cells. Pericytes have been shown to control several aspects of BBB function *in vivo*[13,50] including “barriergenesis” (via release of angiopoietin-1 [51]) and protection against hypoxia-induced BBB disruption [52]. Although our preliminary experiments (*data not shown*) revealed no significant effects of pericytes on BBB tightness, these findings needs to be carefully reproduced under different combinatory approaches of cells (astrocytes/pericytes in different ratios). In addition, functional BBB modulatory effects specific for the endothelium or abluminal astrocytes (e.g., cell polarization) are still poorly understood and need to be further investigated. Another limitation of our study is that we did not attempt to isolate the differentiating effects of shear vs. abluminal cell type. Thus, how astrocytes or vascular smooth muscle influence endothelial cell differentiation under conditions of equal shear stress need to be further investigated. In particular.

## Conclusion

In summary, we have successfully established an *in vitro* dynamic capillary-venule modular system capable of reproducing the physiology and the functional characteristics of multiple segments of the brain vascular network. This innovative model represents a refinement of the DIV-BBB, which has fostered many advances in our understanding of the mechanisms regulating BBB function under normal and disease conditions [53]. The DIV capillary-venule system provides critical, yet unexploited, features to investigate the cerebrovascular response to pathophysiological stimuli. This may help the development and validation of novel CNS therapeutic strategies to reduce the burden of many neurological diseases characterized by poor CNS drug penetration or inflammatory cell interaction with the vascular wall.

## Methods

### Cell Culture

Normal adult human brain microvascular endothelial cells (HBMEC, cat# 1000), human adult astrocytes (HA, cat# 1800) and human brain vascular smooth muscle cells (HBVSMC, cat# 1100) were purchased from ScienCell Research Laboratories (San Diego, CA 92121). HBMEC were expanded in 75 cm^2^ flasks pre-coated with fibronectin (3μg/cm^2^). Growth medium consisted of MCDB 105 (Sigma, Cat# M6395), 10% human AB serum (SIGMA, Cat# S-7148), 15 mg/100 ml of endothelial cell growth supplement (ECGS, Cat.# 1052), 800 units/ml of heparin (Sigma, cat# H3393), 100 units/ml penicillin G sodium and 100 mcg/ml streptomycin sulfate. HA were cultured in Poly-d-Lysine pre-coated flasks (3 μg/cm^2^) with Dulbecco’s modified essential medium (DMEM-F12) supplemented with 2mM glutamine, 5% fetal bovine serum (FBS), 100 units of penicillin G sodium per ml, and 100 μg of streptomycin sulfate per ml.

Smooth muscle cells medium (SMCM; cat# 1101) consists of 500 ml of basal medium, 10 ml of fetal bovine serum (FBS, Cat. No. 0010), 5 ml of smooth muscle cell growth supplement (SMCGS, Cat. No. 1152) and 5 ml of penicillin/streptomycin solution (P/S, Cat. No. 0503). Cell cultures were maintained at 37°C in a humidified atmosphere with 5% CO_2_. Cellular growth was monitored every day by inspection with phase contrast microscopy. To minimize the dedifferentiation process cell cultures were expanded for no more than two cycles.

### DIV-Capillary-Venule setup

Brain primary EC and astrocytes were initially cultured in the DIV-capillary modules, as previously described [54]. However, this iteration of the DIV-BBB consists of 3 hollow polypropylene fibers inside a sealed chamber (the extraluminal space or ECS) accessible by ports. The system allows to reproducing the hemodynamic characteristics (shear stress and blood pressure) of the corresponding vascular segment *in vivo*. Flow was channeled through the bundle of artificial capillaries by gas permeable silicon tubing that allows for the exchange of O_2_ and CO_2_. A pump (CellMax® QUAD Artificial Capillary Cell Culture System, Spectrum Laboratories Inc. CA) feeding the system generates pulsatile high velocity flow and systolic blood pressure (which may range from 80 to 300mmHg) within the silicon tubing, thereby mimicking a pattern of intravascular perfusion comparable to that of physiological blood flow *in vivo*[54]. The luminal surface of the hollow fiber was pre-coated with 3 μg/cm^2^ of fibronectin to facilitate endothelial cells adhesion. The abluminal surface was instead pre-coated with 3 μg/cm^2^ of poly-D-lysine to allow for astrocytes adhesion. The fibers used for capillary vs. venule modules were the same. TEER was measured by a set of electrodes connected to the TEER readout apparatus (Flocel, Inc.).

Endothelial cells were first inoculated into the luminal compartment and allowed to adhere under static conditions over a 24-hr period. In order to achieve higher levels of cell attachment, the flow path was temporarily canalized through the extra-capillary space. Astrocytes were seeded on the abluminal surface of the fibers 24 hours after the initial loading of the endothelial cells. Following astrocytes seeding, the intraluminal flow was re-established and the vascular endothelium was initially exposed to a low level shear stress (1 dyne/cm^2^) for 24 hours.

The venules module consisted of 19 hollow fibers (instead of 3). The higher number of artificial capillaries allowed us to mimic the intramural pressure and vascular shear stress found in the corresponding cerebrovascular segment [42] once the module was serially connected (downstream) to the capillary unit (see Figure 1A and B). The venular hollow fibers were pre-coated (lumen and ablumen) with fibronectin (3μg/cm^2^) to facilitate the adhesion of endothelial cell (in the lumen) and that of HVSM (ablumen). The DIV-venules was then separately exposed to an initial intraluminal shear stress of 1 dyne/cm^2^ for 24 hours prior attachment to the capillary module. Flow was then gradually adjusted to reach the physiological levels of shear stress and intramural pressure *in vivo*.

### TEER measurement

Capillary and venules vascular integrity was monitored in real time by a computer-controlled TEER measurement system (Flocel Inc., Cleveland, OH 44103). This device utilizes electronic multiplexing to reliably measure the TEER in multiple cartridges/modules in quick succession [54,55]. The device interfaced directly to a PC computer via Universal Serial Bus (USB). As previously described, (see also vendor site) the system applies an excitation voltage (0.06 V) across the excitation electrodes inserted in each cartridge in the luminal and extraluminal compartments. A microcontroller computes the resistivity and capacitance per cm^2^ of the vascular bed from physical parameters. The values of capacitance are automatically calculated by comparison of the voltage and current waveforms. TEER was measured continuously from the initial setup throughout the course of each experiment. Previous work in our laboratory [40,55,56] showed a direct (inverse) relationship between TEER and vascular permeability.

### Drug Permeability in capillary and venules modules: uptake of [^14^C]-phenytoin, [^14^C]-diazepam and [^3^H]-sucrose

Boluses (0.5 ml each) of the radioactive tracers [^3^H]-sucrose (Amersham, Piscataway – NJ, Cat# TRA-332), [^14^C]-phenytoin (PerkinElmer, Boston – MA, Cat# NEC-246), and [^14^C]-diazepam (Amersham, Piscataway – NJ, Cat# CFA-591) were injected upstream (before the flow enters the capillary module) into the lumen as described previously [25]. The diffusion of the compounds through the vascular bed into the extracapillary space of each segment of the DIV capillary-venules system was monitored over time while maintaining 1 mL/min intraluminal perfusion rate. A total of 1 μCi per compound was used. Samples were simultaneously taken from the lumen and the ECS (100 μL each) of each module as previously described [25], at time zero (immediately after the injection) and at 1, 5, 10, 15, and 20 min post injection. The luminal samples were collected downstream of each module (where the media leave the cartridge to enter the silicon tubing). Samples were then introduced into vials with 4 mL of Ready Protein Beckman scintillation cocktail (Packard Ultima Gold, ECN, Costa Mesa, CA, USA). Radioactivity was counted with an LS 6500 scintillation counter (Beckman). Permeability across the vascular bed of each module was calculated by graphical integration of drug concentration in the corresponding lumen and ECS over 20 min. Permeability for a given compound in “single pass” experiments was calculated as described elsewhere [25]. In brief, the permeability values were obtained by integrating the area under the ECS and lumen data points (AUC) according to the final equation described below.

In this equation (derived from Fick’s Law) **C**_**ECS**_ (**t**) and **C**_**ECS**_ (**0**) are the extraluminal space concentrations of the compound **x**, at time zero (0) and time (**t**) = 20 minutes. This time interval was chosen to minimize the contribution of drug reperfusion. **V**_**ECS**_ is the volume of the abluminal space (1.33 and 1.15 cm^3^ in the capillary and venule modules respectively); **A** is the capillary surface area (= 2.1 and 13.5 cm^2^ in the capillary and venule modules respectively). To obtain the P value in cm/sec we have divided by 60.

Note that the integral of luminal and ECS values vary only as function of time since the driving force for drug transfer is not (in this equation) concentration dependent. Furthermore, the diffusion of the drug is independent from the dimension of the units of measurement and the same permeability values (in cm/sec) is obtained whether using molar concentration (M) or counts per minute (cpm) units. The permeability results for each compound were then compared to the octanol-water partition coefficient (XLogP) [57] reported for the specific substance (PubChem) to establish a comparative relation between permeability and lipophilicity.

### Pressure analysis

Intramural pressure and waveform geometry of flow passing through each module (capillary and post-capillary) were assessed by four-channel BioTRans2 pressure amplifier (Flocel Inc., Cleveland, OH 44103) connected to 4 independent BioTrans2 pressure transducers. Each transducer was attached to the corresponding inlet and outlet intraluminal sampling ports (ILS port) of each module (see Figure 1A). After a fixed period of 2 minutes (necessary to stabilize the pressure fluctuation), the pulsatile flow patterns were measured and recorded for 60 seconds. The system was connected to an Axon Digitizer (Digidata1322A; Molecular Devices, Inc. Sunnyvale, CA 94089) that functioned as an active interface between the four-channel BioTRans2 pressure amplifier and the computer used for data acquisition.

### Glucose and lactate measurement

Glucose consumption and lactate production were determined via a dual-channel immobilized oxidase enzyme analyzer (YSI 2700 SELECT; YSI Inc., Yellow Springs, OH) in medium samples collected from the capillary and venule modules. Daily assessment of the oxidase enzyme membrane integrity was made according to manufacturer recommendations. The sampling protocol was set to recalibrate the machine every six samples as multiple samples were analyzed in the same run. Samples were kept frozen after being collected and processed later simultaneously.

### BBB “opening” by hyperosmolar mannitol

Infusion of 2 ml of growth medium containing mannitol (1.6M) was used to osmotically open the endothelial layer forming the vascular bed of the capillary and post-capillary segments respectively [28]. The mannitol solution was prepared under sterile conditions and injected intraluminally at a perfusion rate of 1 ml/min (total perfusion time was 120 sec). TEER was monitored in real time in both capillary and venule modules for the duration of the experiment to assess for loss of vascular integrity (opening) and recovery. Osmotic opening via hyperosmolar mannitol injections is a clinical procedure used to facilitate the passage of chemotherapic drugs across the BBB into the CNS for the treatment of malignant brain tumors [58].

#### Statistical analysis

For parametric variables (e.g., TEER levels, glucose consumption, lactate production), differences between populations were analyzed by ANOVA. P values <0.05 were considered statistically significant. Repeated measure ANOVA was performed by JPM software of time-lapse experiments. No differences in significance were found compared to one way ANOVA. Bonferroni analysis was used to account for comparisons of multiple parameters among groups. Based upon previous experience each experiment was repeated in triplicate. This was sufficient to demonstrate statistical significance for positive findings.

## Competing interests

Dr. Janigro has reported the following financial relationships with the companies listed below which may be perceived to bias this work.

**Royalty Payments**. Dr. Janigro has the right to receive royalty payments for inventions or discoveries related to Flocel Inc. He is the founder of Flocel, Inc. which has a commercial interest in flow-based dynamic model of the BBB. The research detailed herein has been performed in accordance to the Cleveland Clinic Foundation COI and a COI approved monitoring plan.

**Equity**. Dr. Janigro owns stocks in Flocel Inc.

Dr. Cucullo has reported the following financial relationships with the companies listed below.

**Equity**. Dr. Cucullo owns stocks in Flocel Inc.

## Authors’ contributions

LC and DJ conceived and supervised the study, elaborated its design. LC drafted the manuscript. LC and DJ also performed the rheological measurements and the statistical analysis. MH established the DIV modules and carried out the experiments. WT carried out the data analysis with LC and provided substantial support in the editing of the manuscript. All authors read and approved the final manuscript.
